# The synthesis of highly active carbon dot-coated gold nanoparticles *via* the room-temperature *in situ* carbonization of organic ligands for 4-nitrophenol reduction†

**DOI:** 10.1039/d0ra02048f

**Published:** 2020-05-21

**Authors:** Yue Zhu, Juan Du, Qianqian Peng, Fengyi Wang, Jing Hu, Yongsong Luo, Abdulmohsen Ali Alshehri, Khalid Ahmed Alzahrani, Baozhan Zheng, Xuping Sun, Dan Xiao

**Affiliations:** Department of Chemistry, Sichuan University Chengdu 610064 Sichuan China zhengbaozhan@scu.edu.cn; Institute of Fundamental and Frontier Sciences, University of Electronic Science and Technology of China Chengdu 610054 Sichuan China xpsun@uestc.edu.cn; Chemistry Department, Faculty of Science, King Abdulaziz University P.O. Box 80203 Jeddah 21589 Saudi Arabia

## Abstract

Due to the serious pollution issue caused by 4-nitrophenol (4-NP), it is of great importance to design effective catalysts for its reduction. Here, a novel and simple strategy was developed for the synthesis of carbon dot-decorated gold nanoparticles (AuNPs/CDs) *via* the *in situ* carbonization of organic ligands on AuNPs at room temperature. The enhanced adsorption of 4-NP on CDs *via* π–π stacking interactions provided a high concentration of 4-NP near AuNPs, leading to a more effective reduction of 4-NP.

## Abstract 2

Due to the serious pollution issue caused by 4-nitrophenol (4-NP), it is of great importance to design effective catalysts for its reduction.

## Graphical abstract

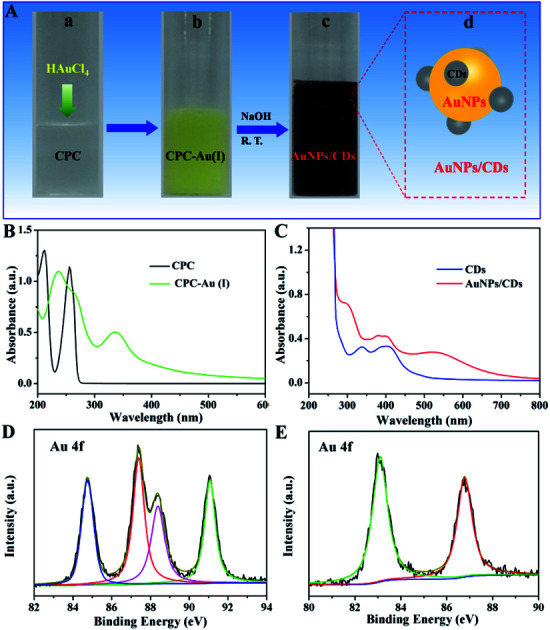

With the rapid development of the global economy and the continuous progress of the society, environmental problems, especially water pollution caused by nitroaromatic compounds, have become increasingly serious during the last decade.^1,2^ As one of the toxic phenolic pollutants, 4-nitrophenol (4-NP) is usually found in the wastewater discharged from many chemical industries, which causes severe environmental issues and also a serious toxic effect on the living organisms.^3–6^ Hence, 4-NP has been classified as a priority toxic pollutant by the US Environmental Protection Agency.^7^ In contrast, 4-aminophenol (4-AP), a product of 4-NP, is less toxic and an important intermediate that can be applied in many fields.^8^ Therefore, developing a novel method to effectively convert hazardous 4-NP to nontoxic 4-AP is urgent for solving the environmental issues.^9^

It has been reported that 4-NP can be reduced to 4-AP by borohydride ions (BH_4_^−^) enabled by catalysts, which plays an important role in this process. Among various catalysts, noble-metal materials (especially Au) have been demonstrated to be effective catalysts and are still the main catalysts used for 4-NP reduction.^10–13^ In any heterogeneous catalysis process, the catalytic reaction must occur on the surface of the catalyst.^14–16^ Therefore, the adsorption of reagents on the catalyst surface is a vital process before the chemical reaction. Recently, the efficient adsorption of reagents with aromatic rings on π-rich supports has been proved based on π–π interactions.^17–19^ As an aromatic compound, 4-NP is also a π-rich molecule in nature, and its adsorption on a carbon-based catalyst by these π–π stacking interactions has also been demonstrated.^20,21^ By decorating metal nanoparticles (MNPs) on these supports, the catalytic activity of MNPs toward the reduction of 4-NP to 4-AP by NaBH_4_ has also been effectively enhanced *via* a synergistic effect.^22,23^ Based on this, various π-rich carbon materials like carbon fibers,^24^ carbon nanotubes,^25^ graphene oxide,^26^ reduced graphene oxide,^27^ and graphitic carbon nitride^28^ have been utilized as supports to prepare metal/carbon-based catalysts with enhanced catalytic activity. However, these methods suffer from drawbacks such as a multiple-step preparation process, prior functionalization, high cost and low yields, limiting their wide practical applications.^29,30^ More importantly, as described previously, the reduction of 4-NP mainly occurs on the AuNP surface;^31^ thus, we can speculate that anchoring smaller π-rich carbon materials on the AuNP surface may lead to higher catalytic activity for the 4-NP reduction. Carbon dots (CDs) with the structure of sp^2^ carbons^32,33^ can adsorb 4-NP *via* π–π stacking, with the additional advantages of excellent stability and smaller size.^34^ Thus, the catalytic activity of AuNPs for 4-NP reduction can be improved by the surface decoration of CDs, which, however, has not been reported before.

Herein, a convenient and simple method was proposed to synthesize CD-decorated AuNPs (AuNPs/CDs) *via* the room-temperature *in situ* carbonization of cetylpyridinium chloride monohydrate (CPC) pre-adsorbed on AuNPs as an organic ligand. This AuNP/CD hybrid was demonstrated to have higher catalytic activity for the reduction of 4-NP, which was about 2.7-fold higher than that of AuNPs. The improved catalytic activity of AuNPs/CDs can be attributed to the synergistic effect between AuNPs and CDs.

According to previous research, CPC can be carbonized to carbon dots in the presence of NaOH.^35,36^ Inspired by this, we used CPC as both the capping and reducing agent to synthesize AuNPs in the presence of NaOH, and the synthesis process is displayed in Fig. 1A. After adding HAuCl_4_ into an aqueous solution of CPC, yellow suspended solids were generated quickly (Fig. 1A(a) and (b)), which should be attributed to the formation of CPC-Au(i) between AuCl_4_^−^ and CPC. Fig. 1B shows the UV-vis spectra of the aqueous solution of CPC before and after the addition of AuCl_4_^−^. The obvious absorption peak at 340 nm (green) should be due to the generated CPC-Au(i). The generated Au(i), confirmed by the XPS analysis results (Fig. 1D), can originate from the reduction of Au(iii) by CPC because there are no extra reducing agents present in this system. After adding NaOH into the above-mentioned CPC-Au(i) solution at room temperature (Fig. 1A(b) and (c)), the color of the solution gradually changed to dark red (Fig. 1A(c)). The UV-vis spectrum of the above-mentioned mixture solution was then recorded. The absorbance between 280 and 450 nm proved the formation of CDs due to the carbonization of CPC under alkaline conditions;^35^ moreover, the obvious absorption peak at 520 nm (Fig. 1C (red)), corresponding to the surface plasmon resonance (SPR) of AuNPs,^37^ indicated the formation of AuNPs in this process. The formation of AuNPs could be attributed to the further reduction of CPC-Au(i) after the addition of NaOH. XPS analysis was also performed to investigate the oxidation state of the Au species in the process of AuNP synthesis. The high-resolution Au 4f XPS spectra shown in Fig. 1D and E indicate the presence of different Au species in these two samples. For CPC-Au(i) (Fig. 1D), the four evident peaks at 84.8, 88.4 eV and 87.4, 91.1 eV were assigned to the Au 4f_7/2_ and Au 4f_5/2_ signals,^38^ respectively, indicating the co-existence of nonmetallic Au^+^ and Au^3+^ in this sample.^39^ However, for AuNPs/CDs (Fig. 1E), two typical binding energies at 86.8 eV and 83.1 eV, attributed to the binding energies of metallic Au(0),^40^ were clearly observed, indicating the successful reduction of Au(iii) and/or Au(i) to Au(0) in the presence of NaOH.

**Fig. 1:**
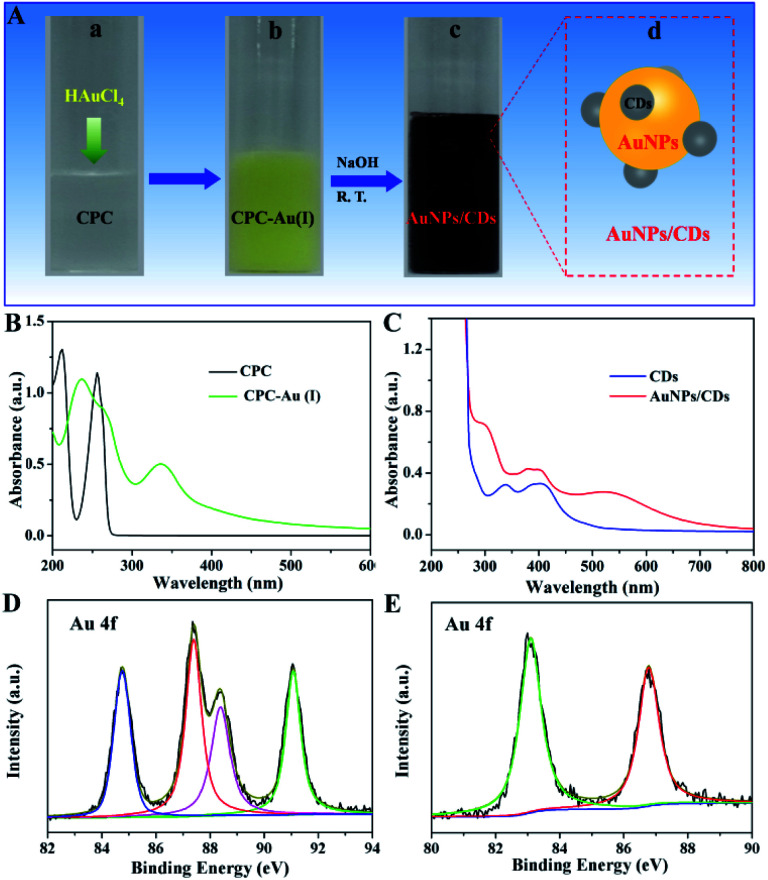
(A) Diagram for the synthesis of AuNP/CD nanocomposites at room temperature. (B and C) The UV-vis absorption spectra of CPC (black), CPC-Au(i) (green), CDs (blue) and AuNPs/CDs (red) in aqueous solutions, respectively. The high-resolution XPS spectra of Au 4f in CPC-Au(i) (D) and AuNPs/CDs (E).

Fig. 2A displays the TEM image of the synthesized AuNPs/CDs. From the image, it can be seen that the monodispersed AuNPs/CDs are spherical, with an average size of 3.5 ± 0.8 nm and a standard deviation of the particle sizes of 22.9% (Fig. S1†). This relatively small size should be attributed to the use of CPC as a capping agent, which can effectively control the growth of AuNPs. Fig. 2B shows the HRTEM image of AuNPs/CDs; the characteristic lattice spacing of 2.06 Å can be attributed to the (200) plane of face-centered cubic (fcc) gold,^41^ which also indicates the successful synthesis of Au nanocrystals under these conditions. In addition, the presence of CDs on the AuNP surface can be clearly observed (Fig. 2B and C), and the Raman peak of AuNPs/CDs between 1100 and 1800 cm^−1^ (Fig. S2†) also indicates the presence of carbon dots on AuNPs.^42^Fig. 2D shows the XRD patterns of CDs (black) and AuNPs/CDs (red). There is no diffraction peak for CDs, indicating the amorphous structure of CDs. However, the obvious peaks at 38.9°, 44.8°, 65.1° and 78.1° (red) for AuNPs/CDs, assigned to the diffraction from the (111), (200), (220) and (311) planes, respectively, of fcc Au crystals,^43^ also prove the successful formation of Au nanocrystals and agree well with the results of TEM. Fig. 2E displays the FT-IR spectra of AuNPs/CDs (red) and CDs (black). Similar groups can also be found on both CDs and AuNPs/CDs, indicating the presence of CDs on the AuNP surface. In a word, the results described above not only prove the successful synthesis of AuNPs, but also the structure of CDs decorated on the AuNP surface based on the *in situ* carbonization of CPC at room temperature.

**Fig. 2:**
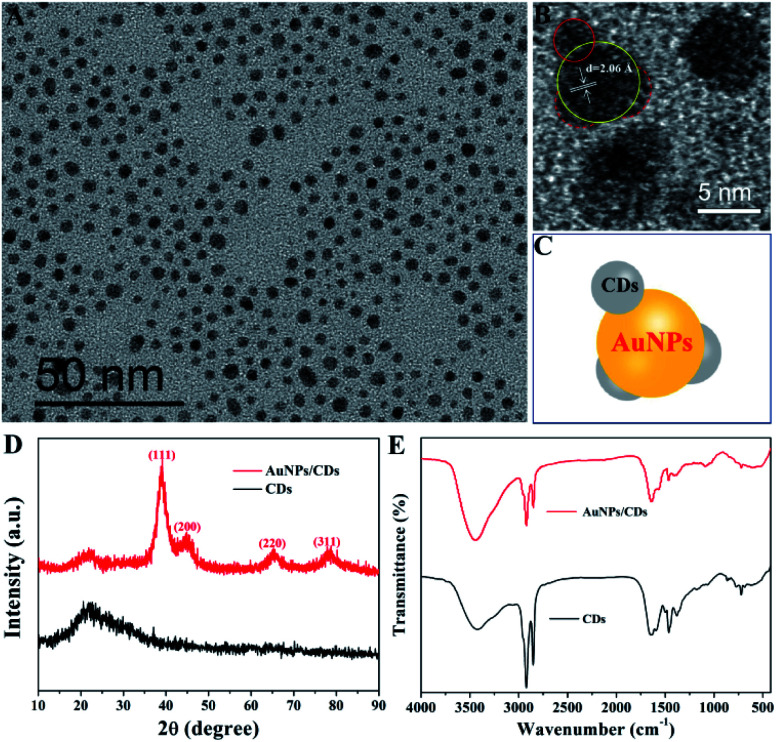
(A) TEM and (B) HRTEM images of AuNPs/CDs. (C) The structure of AuNPs/CDs. (D) XRD profiles and (E) FT-IR spectra of CDs (black) and AuNPs/CDs (red).

The reduction of 4-NP to 4-AP with an excess amount of NaBH_4_ was carried out to quantitatively evaluate the catalytic properties of AuNPs/CDs. In the absence of the catalyst, a strong absorbance peak at 400 nm was observed for the mixture of 4-NP and NaBH_4_, which was attributed to the 4-NP ions under alkaline conditions.^44^ After adding the catalysts CDs and AuNPs/CDs into the reaction system, the reduction process was monitored by measuring the time-dependent absorption spectra of the mixed reaction solution. As shown in Fig. 3A, the nearly unchanged absorption even after one hour when only CDs were present as the catalyst indicates the non-active nature of CDs for 4-NP reduction. However, when we used AuNPs/CDs as the catalyst, the absorbance intensity of 4-NP at 400 nm decreased quickly as the reaction time was extended, and this was accompanied by the appearance of an absorbance peak of 4-AP at 300 nm (Fig. 3B), indicating the higher catalytic activity of AuNPs/CDs for the reduction of 4-NP to 4-AP by NaBH_4_. This result also indicates that the reduction of 4-NP should be attributed to the catalysis of AuNPs in AuNPs/CDs due to the non-active CDs. It should be noted that the reduction of 4-NP by NaBH_4_ could be completed within 10 minutes, with the observation of fading and ultimate leaching of the yellow-green color of the reaction mixture in the aqueous solution. However, a longer reaction time (more than 18 minutes) was required to achieve the full reduction of 4-NP under similar conditions with AuNPs alone (synthesized with trisodium citrate; see ESI† for preparation details) as the catalyst (Fig. 3C). Fig. 3D shows the absorption changes of the solutions at 400 nm in the presence of different catalysts, and the quicker decrease in absorption indicates the higher catalytic activity of AuNPs/CDs than that of AuNPs. Since the concentration of BH_4_^−^ was constant during the catalytic reaction and much higher than that of 4-NP, the rate constants could be evaluated by the pseudo-first-order kinetics using ln(*C*_*t*_/*C*_0_) = −*kt*, where *k* is the apparent first-order rate constant; its value estimated directly from the slope of the straight line can be used to evaluate the catalytic activity of a catalyst.^45^ As shown in Fig. 3E, a good linear relationship of ln(*C*_*t*_/*C*_0_) *versus* the reaction time (*t*) is observed for the two catalysts; the rate constant (*k*) values were calculated as 1.83 × 10^−4^ min^−1^, 0.11 min^−1^ and 0.30 min^−1^ for CDs, AuNPs and AuNPs/CDs, respectively. These results clearly demonstrated the higher catalytic activity of the AuNPs/CDs composites, which was about 2.7-fold higher than that of AuNPs. In addition, the AuNPs/CDs catalyst exhibited better/comparable catalytic activity compared to/to that of previously reported modified gold catalysts in terms of the time needed for the reduction reaction and rate constant (Table S1†), demonstrating its potential applications in catalysis.

**Fig. 3:**
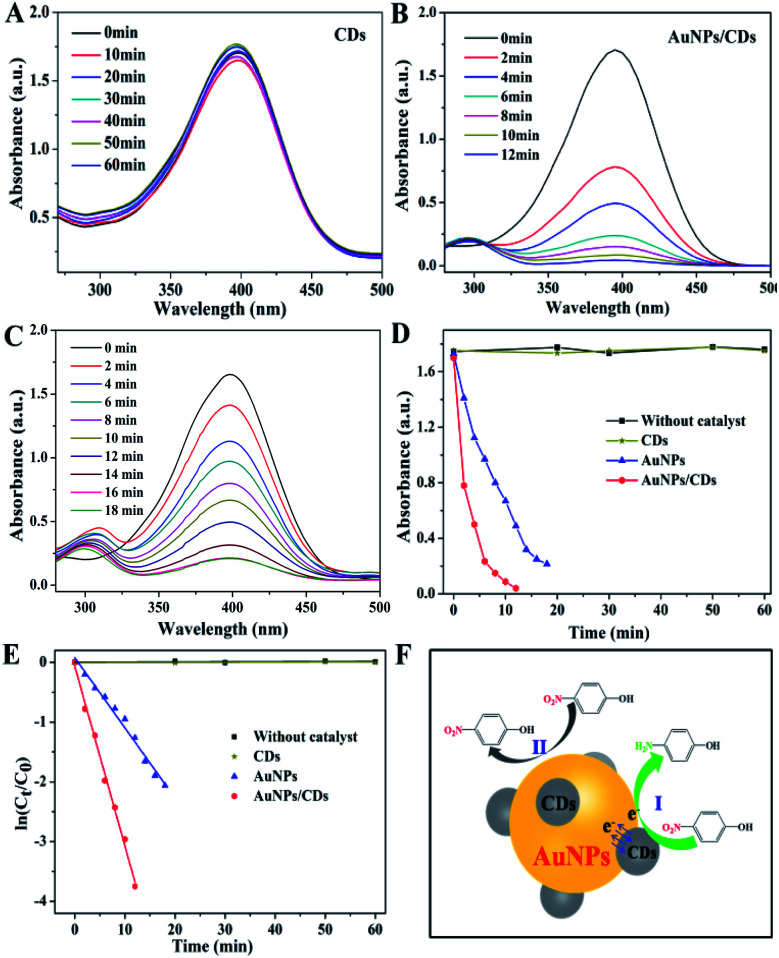
The UV-vis absorption spectra for the reduction of 4-NP by CDs (A), AuNPs/CDs (B) and AuNPs (C). The plots of absorbance (D) and ln(*C*_*t*_/*C*_0_) (E) *versus* reaction time for the catalytic reduction of 4-NP based on different catalysts. (F) The mechanism for the enhanced catalytic activity of AuNPs/CDs for 4-NP reduction.

Based on the experimental results and analysis mentioned above, the enhanced catalytic activity of AuNPs/CDs for 4-NP reduction should be attributed to the presence of CDs on AuNPs, which leads to a synergistic effect between AuNPs and CDs. This synergistic effect plays an active part in catalysis and therefore enhances the catalytic activity of AuNPs/CDs, which can be explained as follows: as a π-rich molecule, 4-NP can be adsorbed onto the surface of CDs *via* π–π stacking interactions.^20,23^ In addition, the adsorption of 4-NP on AuNPs/CDs can be demonstrated by the decreased absorbance of the 4-NP solution after adding AuNPs/CDs in the presence of NaOH instead of NaBH_4_ (Fig. S3†). This endows AuNPs/CDs with anchoring sites for the adsorption of 4-NP, and such strong adsorption results in a higher concentration of 4-NP near the surface of AuNPs,^20^ leading to efficient contact between them and therefore speeding up the catalytic process (Fig. 3F(I)). In contrast, for AuNPs without the presence of CDs, 4-NP must collide with AuNPs by chance and must remain in contact for the catalysis to proceed; or else, 4-NP will pass back into the solution and the reaction cannot occur (Fig. 3F(II)) until it reaches the AuNP surface again.^46^ This can also be demonstrated by the increased reaction rate constant for the 4-NP reduction with more catalysts present in the reaction system (Fig. S4†). In addition, due to the excellent electron acceptor and donor properties of CDs,^47,48^ which can obtain/give excess electrons from/to AuNPs (Fig. 3F), like a reservoir of electrons, the electron transfer from CDs to AuNPs increases the local electron concentration and maintains AuNPs in an electron-enriched state,^49^ facilitating the uptake of electrons by the 4-NP molecules.

In summary, a nanocomposite of AuNPs/CDs was successfully synthesized *via* the room-temperature *in situ* carbonization of CPC as a capping agent for AuNPs. As a catalyst for 4-NP reduction, this composite showed superior catalytic performances to AuNPs, which could be rationally attributed to the enhanced adsorption of 4-NP on CDs providing a high concentration of 4-NP near AuNPs for a more effective reduction of 4-NP. This work not only offers an attractive catalyst material for 4-NP reduction, but also opens an exciting new avenue for the rational design and development of CD/metal nanostructure hybrids for various applications.

## Conflicts of interest

There are no conflicts to declare.

## Supplementary Material

RA-010-D0RA02048F-s001
