# Supplementary material for: The synthesis of highly active carbon dot-coated gold nanoparticles via the room-temperature in situ carbonization of organic ligands for 4-nitrophenol reduction
Source: RSC Adv. 2020 May 21;10(33):19419–24. doi: 10.1039/d0ra02048f (PMC9054074; doi:10.1039/d0ra02048f)
Supplement: RA-010-D0RA02048F-s001 [file RA-010-D0RA02048F-s001.pdf]

Electronic Supplementary Material (ESI) for RSC Advances.
This journal is © The Royal Society of Chemistry 2020

Electronic Supplementary Information

Experimental section

Materials

Cetylpyridinium chloride monohydrate (CPC) and chloroauric acid tetrahydrate ($HAuCl_4{\cdot}4H_2O$) were purchased from Alfa Aesar China Chemical Co., Ltd (Shanghai, China). 4-Nitrohenol (4-NP) was from Sigma-Aldrich (St Louis, MO, USA). Trisodium citrate, sodium hydroxide (NaOH), ethanol, sodium borohydride ($NaBH_4$) were bought from Chengdu Kelong Chemical Co., Ltd (Chengdu, China). The water (≥ 18.2 M Ohm cm) used in all experiments was from a Millipore Milli-Q-RO4 water purification system (Bedford, MA, USA). All chemicals were analytic grade reagents, and used without further purification.

Synthesis of AuNPs/CDs, CDs and AuNPs

The gold nanoparticles (AuNPs) were one-step synthesized in aqueous solution with a simple and convenient method by directly adding $HAuCl_4$ into CPC aqueous solution with the presence of NaOH. Typically, $HAuCl_4$ aqueous solution (2 mL, 25 mM) was added into 10 mL CPC aqueous solution (25 mM) with stirring, and yellow precipitate was formed quickly at room temperature. After that, 2 mL NaOH aqueous solution (2 M) was added into the above mixture, and the color of the solution turned to dark wine-red, indicating the formation of AuNPs. After the reaction for 1 hour at ambient conditions, the solution was then centrifuged and washed with ethanol for several times, and carbon dots (CDs) decorated AuNPs can be obtained. The CDs was synthesized with the same process without the addition of $HAuCl_4$ in CPC solution.

AuNPs was synthesized using trisodium citrate as capping and reducing agents. In a typical synthesis, a solution of 4.0 mM sodium citrate in water (100 mL) was heated in a 250 mL three-necked round-bottomed flask under vigorous stirring. When it was boiling, 2.00 mL of $HAuCl_4$ (25 mM) was then added into the above solution. Keeping the solution boiling for 30 min, the reaction was finished by cooling down and AuNPs was obtained.

Characterizations

The UV-Vis adsorption spectra of sample were measured on a Hitachi U-2900 UV-Vis absorption spectrophotometer (Tokyo, Japan). X-ray diffraction (XRD)

measurements were carried out on a Tongda TD-3500 X-ray powder diffractometer (Liaoning, China) with Cu Kα radiation (λ=0.154 nm). The transmission electron microscopy (TEM) images were acquired on a Hitachi H-8100EM transmission electron microscope (Tokyo, Japan) with an accelerating voltage of 200 kV using carbon coated copper grids. The Fourier transform infrared (FT-IR) spectra were characterized on a Thermo Scientific Nicolet 6700 FT-IR spectrometer (Sugar Land, TX, USA). The X-ray photoelectron spectra (XPS) were recorded on a Kratos AXIS Ultra DLD X-ray Photoelectron Spectroscope (Kratos, UK) using Mg as excitation source.

Investigation catalytic properties of AuNPs/CDs, CDs and AuNPs.

The catalytic properties of synthesized AuNPs/CDs was investigated by the reduction of 4-Nitrophenol (4-NP) with sodium borohydride ($NaBH_4$), all catalytic reactions were carried out at room temperature. Typically, in a 100 mL beaker, 5 mL freshly prepared $NaBH_4$ solution (600 mM) was mixed with 10 mL of 4-NP aqueous solution (0.5 mM), and the final reaction volume was diluted to 50 mL with distilled water. The catalysts (AuNPs/CDs) were then added into the above solution under continuous stirring. For monitoring the reaction process, 1 mL of reaction solution was taken out with a filter at set intervals, and then injected into a quartz cuvette immediately for the measurement of adsorption on the UV-vis absorption spectrometer. In addition, to study the catalytic activity of CDs and AuNPs for the 4-NP reduction, equal amount of CDs and AuNPs instead of AuNPs/CDs were added into the reaction system with the same procedure as mentioned above.

Table S1. Comparison of different gold based nanocatalysts used for 4-NP reduction.

**Catalyst**	**Catalytic duration (min)**	**Rate constant (k) ($\times 10^{-3}$ s^{-1})**	**Ref.**
AuNPs/GO	18	2.03	1
Au/grapheme hydrogel	12	3.17	2
Au-PDA/RGO	16	2	3
Au-dendrimer	18	2.41	4
Au/OMS composite	57	0.97	5
Av-AuNPs	20	1.55	6
AuNPs	17	2.6	7
Fe_3O_4@COF- Au	20	3.7	8

Au/g-C_3N_4-1~6	>10	0.6-7.9	9
AuCu (Triangular)	>20	5.2	
AuCu (Hemispherical)	>20	2.6	10
Au (Wulff)	>30	0. 41	
AuNPs/TWEEN/GO	14	4.2	11
AuNPs/TWEEN	32	0.15	
AuNPs alone	18	1.83	This work
AuNPs/CDs	10	5	This work

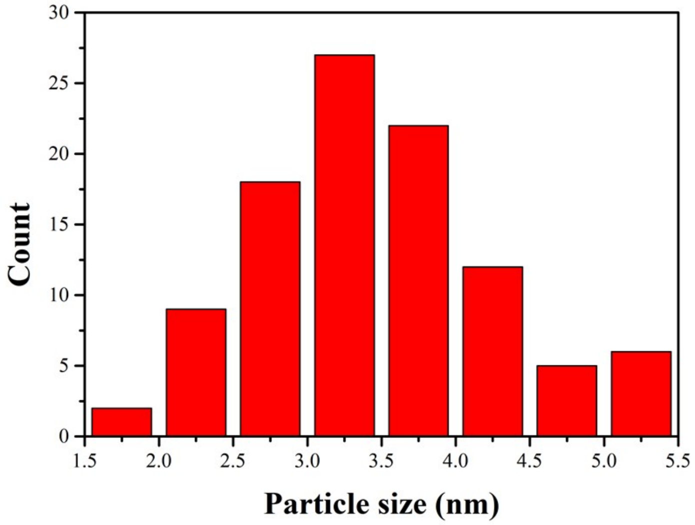

Fig. S1 The size distribution histogram of AuNPs/CDs.

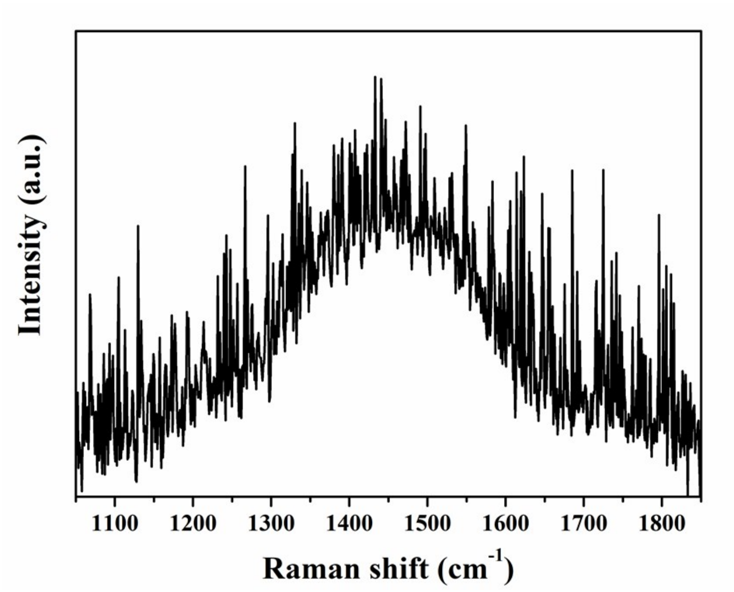

Fig. S2 The Raman spectrum of AuNPs/CDs.

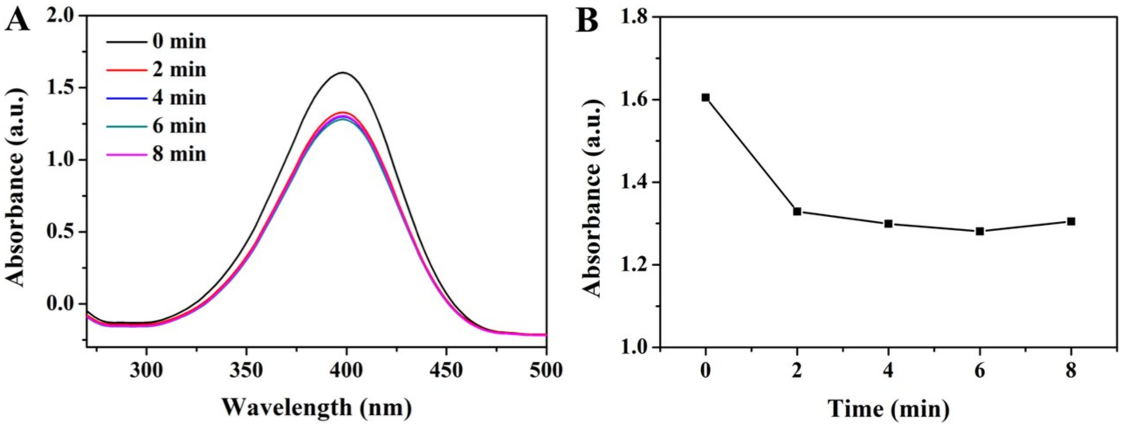

Fig. S3 (A) UV-visible spectra of 4-NP after adding AuNPs/CDs in the presence of NaOH instead of $NaBH_4$. (B) The corresponding changes of absorbance at 400 nm from A.

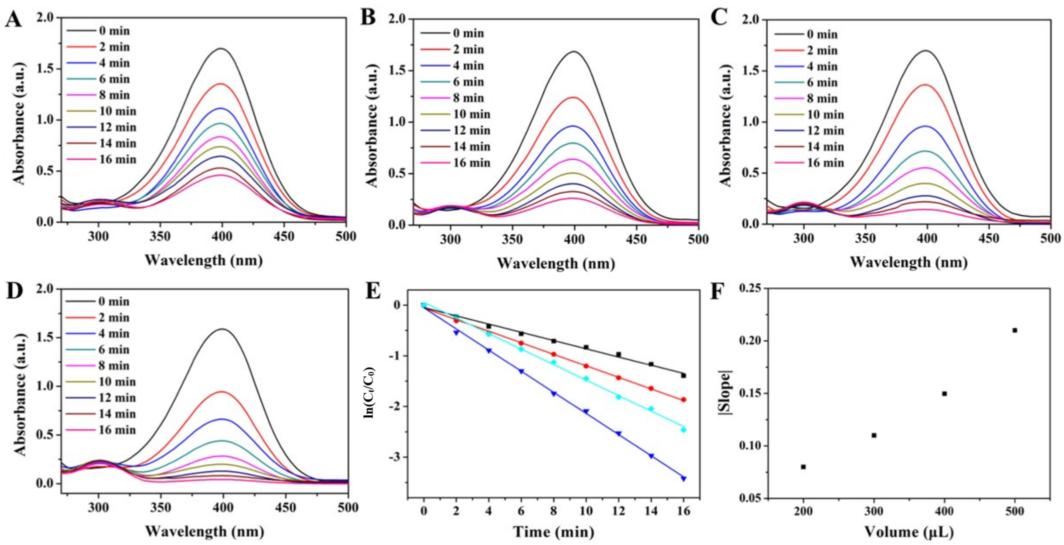

Fig. S4 (A-D) UV-vis spectra of different catalyst amounts (200, 300, 400, 500 μL) and (E) $\ln(C_t/C_0)$ over time and (F) graph of Slope (rate constant) over time.

References.

1. Y. Zhang, S. Liu, W. Lu, L. Wang, J. Tian and X. Sun, In situ green synthesis of Au nanostructures on graphene oxide and their application for catalytic reduction of 4-nitrophenol, *Catal. Sci. Technol.*, 2011, **1**, 1142-1144.
2. J. Li, C. Y. Liu and Y. Liu, Au/graphene hydrogel: synthesis, characterization and its use for catalytic reduction of 4-nitrophenol, *J. Mater. Chem.*, 2012, **22**, 8426-8430.
3. W. C. Ye, J. Yu, Y. X. Zhou, D. Q. Gao, D. A. Wang, C. M. Wang and D. S. Xue, Green synthesis of Pt-Au dendrimer-like nanoparticles supported on polydopamine-functionalized graphene and their high performance toward 4-nitrophenol reduction, *Appl. Catal. B*, 2016, **181**, 371-378.
4. K. Hayakawa, T. Yoshimura and K. Esumi, Preparation of gold-dendrimernanocomposites by laser irradiation and their catalytic reductionof 4-nitrophenol *Langmuir,* 2003, **19,** 5517-5521.

5. J. Wei, H. Wang, Y. Deng, Z. Sun, L. Shi, B. Tu, M. Luqman and D. Zhao, Solvent evaporation induced aggregating assembly approach to three-dimensional ordered mesoporous silica with ultralarge accessible mesopores, *J. Am. Chem. Soc.*, 2011, **133**, 20369-20377.
6. N. K. R. Bogireddy and V. Agarwal, *Persea americana* seed extract mediated gold nanoparticles for mercury(II)/iron(III) sensing, 4-nitrophenol reduction, and organic dye degradation, *RSC Adv.*, 2019, **9**, 39834-39842.
7. X. Q. Wu, X. W. Wu, Q. Huang, J. S. Shen and H. W. Zhang, *In situ* synthesized gold nanoparticles in hydrogels for catalytic reduction of nitroaromatic compounds, *Appl. Surf. Sci.*, 2015, **331**, 210-218.
8. Y. L. Xu, X. F. Shi, R. Hua, R. Zhang, Y. J. Yao, B. Zhao, T. Liu, J. Z. Zheng, G. Lu, Remarkably catalytic activity in reduction of 4-nitrophenol and methylene blue by Fe_3O_4@COF supported noble metal nanoparticles, *Appl. Catal. B,* 2020, **260**, 118142.
9. Y. S. Fu, T. Huang, B. Q. Jia, J. W. Zhu, X. Wang, Reduction of nitrophenols to aminophenols under concerted catalysis by Au/g-C_3N_4 contact system, *Appl. Catal. B,* 2017, **202**, 430-437.
10. M. Hajfathalian, K. D. Gilroy, A. Yaghoubzade, A. Sundar, T. Tan, R. A. Hughes and S. Neretina, Photocatalytic enhancements to the reduction of 4-nitrophenol by resonantly excited triangular gold–copper nanostructures, *J. Phys. Chem. C*, 2015, **119**, 17308-17315.
11. W. Lu, R. Ning, X. Qin, Y. Zhang, G. Chang, S. Liu, Y. Luo and X. Sun, Synthesis of Au nanoparticles decorated graphene oxide nanosheets: Noncovalent functionalization by TWEEN 20 in situ reduction of aqueous chloroaurate ions for hydrazine detection and catalytic reduction of 4-nitrophenol, *J. Hazard. Mater.*, 2011, **197**, 320-326.
